# Consenting for current genetic research: is Canadian practice adequate?

**DOI:** 10.1186/1472-6939-15-80

**Published:** 2014-11-20

**Authors:** Iris Jaitovich Groisman, Nathalie Egalite, Beatrice Godard

**Affiliations:** Omics-Ethics Research Group, Department of Social and Preventive Medicine, School of Public Health, Université de Montréal, C.P. 6128 succ. Centre-ville, Montreal, H3C 3 J7, Canada

**Keywords:** Participants’ protection, Institutional review boards, Next generation sequencing, Secondary use, Recontacting participants, Data sharing, Genetic research, Informed consent, Vulnerable populations, Mental health, Brain disorders

## Abstract

**Background:**

In order to ensure an adequate and ongoing protection of individuals participating in scientific research, the impacts of new biomedical technologies, such as Next Generation Sequencing (NGS), need to be assessed. In this light, a necessary reexamination of the ethical and legal structures framing research could lead to requisite changes in informed consent modalities. This would have implications for Institutional Review Boards (IRBs), who bear the responsibility of guaranteeing that participants are verifiably informed, and in sufficient detail, to understand the reality of genetic research as it is practiced now. Current literature allowed the identification of key emergent themes related to the consent process when NGS was used in a research setting.

**Methods:**

We examined the subjects of secondary use, sharing of materials and data, and recontacting participants as outlined in the Canadian Informed Consent templates and the accompanying IRB instructions for the conduct of genetic research. The research ethics policy applied by the three Canadian research agencies (Tri-Council Policy Statement, 2^nd^ Edition) was used to frame our content analysis. We also obtained IRB-approved consent forms for genetic research projects on brain and mental health disorders as an example of a setting where participants might present higher-than-average vulnerability.

**Results:**

Eighty percent of documents addressed different modalities for the secondary use of material and/or data, although the message was not conveyed in a systematic way. Information on the sharing of genetic sequencing data in a manner completely independent of the material from which it originated was absent. Grounds for recontacting participants were limited, and mainly mentioned to obtain consent for secondary use. A feature of the IRB-approved consent documents for genetic studies on brain and mental health disorders using NGS technologies, offered a complete explanation on sharing material and data and the use of databases.

**Conclusions:**

The results of our work show that in Canada, many NGS research needs are already dealt with. Our analysis led us to propose the addition of well-defined categories for future use, adding options on the sharing of genetic data, and widening the grounds on which research participants could consent to be recontacted.

## Background

There is a constant need to fine-tune the balance between the potential of new biomedical advances and the ethical and legal structures needed to ensure the protection of human subjects involved in research projects [1]. It is becoming clear that identical consent modalities cannot accommodate all scenarios. For instance, recent reports propose reforming the ethical oversight for low-risk randomized research projects, using an abridged consent process, where participants are not required to sign consent forms [2]. In the case of genomic studies, the advent of Next Generation Sequencing (NGS) is changing the previously established balance in the consent process [3–5]. As a result, there is an ongoing discussion about how new IC models and approaches should evolve, with a view to conveying necessary information while buttressing the autonomy of participant decisions [6, 7]. Proposed new models involve the use of parceled out IC for result disclosure, implying an active collaboration between ethics boards, researchers and participants so as to provide options for returning results according to immediate actionability, reproductive significance and personal utility [6]. Another example is the “Informed Cohort” model in which there is continuous interaction between researchers and participants when providing and receiving genetic results, entailing the active use of educational tools [7]. These changes impel Institutional Review Boards (IRBs) to adapt their ethical reviews to a new language and entertain novel concepts in order to better safeguard research participants’ rights without hindering expected scientific advancements. Therefore, IRBs may need to see if their current recommendations to researchers, offered in the form of templates and guiding documents, are apt to offer research participants information that truly reflect the state of genetics research as well as the paths it may yet follow.

Previous analysis on the use of NGS in research and the ethical and social issues surrounding participant protection allowed the identification of several key, emergent themes related to the consent process [8, 9]. Important aspects include the assurance of understanding, by participants, of both the science underlying the protocol and the complex possible outcomes of current genetic research; adequate provision of genetic counseling; confidentiality; specifications of secondary use and sharing of material and data, and the various reasons for frequent contact between researchers and participants, among others. In this manuscript, we discuss the subjects of secondary use (also referred to as future use), sharing of material and data in relation to secondary use, and recontacting participants. These three subjects are interconnected and have in common that in order to be implemented, they require anticipation by, and coordination between, IRBs and researchers; they could be viewed as vague concepts by participants, which makes them harder to explain; and they are directly linked to the principle of respecting privacy. The goal of this study was to determine if current Canadian IC templates and accompanying IRB instructions are consistent with the ethical challenges posed by the rise of NGS technologies in the aforementioned contexts. We used the research ethics policy applied by the three Canadian research agencies (Tri-Council Policy Statement 2^nd^ Edition, hereafter TCPS2) [10] to frame our content analysis. Institutions that receive and administer research funds from these agencies must comply with the TCPS2. Finally, based on earlier research [8, 9, 11] indicating that research participants with brain and mental health disorders might present higher-than-average vulnerability, we examined IRB-approved consent forms for NGS research protocols targeting this population.

## Materials

### Sample collection

In order to evaluate if Canadian IC templates and accompanying IRB instructions address the current challenges of secondary use, sharing and recontacting participants in the framework of genetic research, the collection process consisted of selecting consent templates and guidelines pertaining to genetic/genomic studies. We also included documents produced for the banking of biological materials in the context of a genetic/genomic research project. We collected publicly available consent form templates from Canadian IRBs, various websites (universities, hospitals, research institutes), or by requesting templates from members of the Canadian Association of Research Ethics Boards (CAREB) or IRB personnel, when their contact information was publicly available. We sought to achieve the widest possible Canadian geographical representation. In the course of our study, we obtained IRB-approved consent forms for different NGS research projects studying mental health and brain disorders currently conducted at Canadian institutions.

Sample documents were collected between January and September 2012. At the end of this period, all information was rechecked for currency. Documents that had been modified since the initial collection were updated until the assembly of material was considered complete.

We gathered a total of 60 documents. Thirteen out of the 60 documents were excluded either because of duplication or because they were unrelated to our goals. Our final sample consisted of 47 documents. In addition, we obtained six IRB-approved consent forms for genetic projects using NGS technologies and covering brain and mental health disorders.

## Methods

### Document classification

We chose to work with templates because we considered them accessible tools used to build the consent forms later subjected to IRB evaluation, a reflection of IRB guidelines or policies. The arrangement of documents was not uniform among IRBs, as the following variants partly attest to: ‘Consent Form for Genetic Research’, ‘Primary Consent Form’ with a separate ‘Addendum for Genetic Studies’. Given this divergence, we organized the documents as units of analysis (UAs), in the same way as the IRBs indicated they should be used by researchers. Units of analysis were comprised of individual forms (e.g., consent form template) or a combination of forms (e.g., primary consent template with additional secondary template for genetic research), much as they would be presented to a potential research participant. If an IRB produced one consent template for genetics research with adult participants and a different one for children, we analyzed them as two separate units of analysis: one for adult genetics and another for pediatric genetics. This distinction was drawn to capture possible differences characterizing each population. UAs were classified according to the scope of research, i.e., Cancer Genetics; Pediatric Genetics; Genetics; Pharmacogenomics, etc. (Table 1a).

**Table 1:** **Classification of units of analysis (UAs), headings addressing and options for secondary use**

a: Secondary use: classification of units of analysis (UAs)			Document		Total
			Primary	Primary plus addendum	
No	Scope of research	Generic	1		1
		Genetics	2		2
		Tissue Genetics	1		1
	Total		4		4
Yes	Scope of research	Cancer Genetics	1	2	3
		Cardiovascular Genetics	1	0	1
		Clinical Genomics	1	0	1
		Generic	1	0	1
		Genetics	12	7	19
		Mental Health Genetics	2	1	3
		Pediatric Genetics	2	2	4
		Tissue Genetics – Future	0	1	1
	Total		20	13	33
Total	Scope of research	Cancer Genetics	1	2	3
		Cardiovascular Genetics	1	0	1
		Clinical Genomics	1	0	1
		Generic	2	0	2
		Genetics	14	7	21
		Mental Health Genetics	2	1	3
		Pediatric Genetics	2	2	4
		Tissue Genetics	1	0	1
		Tissue Genetics – Future	0	1	1
	Total		24	13	37
**b: Headings**		**Frequency**	**Percent**	**Valid percent**	**Cumulative percent**
Valid	Banking	1	2.7	2.7	2.7
	Banking for future research	1	2.7	2.7	5.4
	Biobanking for future research, consent	1	2.7	2.7	8.1
	Biobanking for future research, use of samples and data, consent	1	2.7	2.7	10.8
	Biobanking for future research, use of samples, consent, access to samples, risk	1	2.7	2.7	13.5
		2	5.4	5.4	18.9
	Consent	2	5.4	5.4	24.3
	Consent, subject care	1	2.7	2.7	27.0
	Description of research, consent	1	2.7	2.7	29.7
	Description of research, consent, use of samples	1	2.7	2.7	32.4
	Description of the research, consent, future research	1	2.7	2.7	35.1
	Future contact/future research/other use	1	2.7	2.7	37.8
	Future use, biological specimens	1	2.7	2.7	40.5
	Introduction, optional studies	1	2.7	2.7	43.2
	Invitation to participate	1	2.7	2.7	45.9
	Main title, introduction, consent	1	2.7	2.7	48.6
	Not applicable	5	13.5	13.5	62.2
	No heading	1	2.7	2.7	64.9
	Optional studies, confidentiality	1	2.7	2.7	67.6
	Options to participants	2	5.4	5.4	73.0
	Other research	2	5.4	5.4	78.4
	Other Research, consent	1	2.7	2.7	81.1
	Procedures	1	2.7	2.7	83.8
	Purpose of study, study procedures	1	2.7	2.7	86.5
	Sample collection, project description, consent	1	2.7	2.7	89.2
	Secondary use	1	2.7	2.7	91.9
	Use of samples	1	2.7	2.7	94.6
	Withdraw	2	5.4	5.4	100.0
	Total	37	100.0	100.0	
**c: Options for secondary use**		**Frequency**	**Percent**	**Valid percent**	**Cumulative percent**
Valid	Determined by consenting to participate in the study	5	13.5	13.5	13.5
	NA	5	13.5	13.5	27.0
	No future use plus other options	8	21.6	21.6	48.6
	Other research	1	2.7	2.7	51.4
	Various options	18	48.6	48.6	100.0
	Total	37	100.0	100.0	

Following an early stage of examining the material, we determined that the themes of our analysis were not always present in all documents. Thereafter we searched for other texts issued by each corresponding institution so that their approach to the issues dealt with in this manuscript was reflected as completely as possible. In other words, before stating that a subject was not dealt with by an IRB, we made sure that it was not present in any of the material produced by that body. Thus, UAs were composed of one or more consent form templates (depending on, for instance, whether an IRB worked with a single consent form for genetic studies or a general consent form and a secondary, optional one, for genetic studies), whereas in some cases, UAs were composed of a template(s) and an additional document (i.e., guidelines).

We examined a total of 37 UAs that resulted from combining 47 different template documents without disclosing the institutions from which those were obtained.

Due to their limited number, IRB-approved consent forms for studies involving the genetics of brain or mental health disorders were analyzed as a distinct group, so as to avoid any possible identification of the researchers involved, and also because they did not lend themselves as well to comparison.

### Data analysis process

We applied content analysis to the UAs as previously described [12, 13]. Content analysis is a general term for a number of different strategies used to analyze texts. It seeks to describe the characteristics of a document’s content by examining, for instance, what is being said, to whom, and to what effect. It allows for both quantitative and qualitative analysis of data [14, 15].

Each document was first read so as to identify key themes and then read again and coded to obtain the sense of the whole and to identify additional key themes. Finally, all key themes were subdivided into any subthemes that could emerge. This analysis was informed by issues linked to the consent process in the context of NGS technologies (i.e., whole genome sequencing, exome sequencing), issues that were brought to the fore in recent analyses [8, 9]. We evaluated the current ethical issues in need of deeper analysis in light of the application of the newest genomic technologies in research and the ensuing implications for individuals and social groups.

The reliability and validity of the findings were strengthened by having two individuals with different fields of expertise (IJG, molecular biology; NE, bioethics) analyzing the IC documents separately to code them. The next stage of data analysis consisted of open coding, collecting codes under potential themes and subthemes, comparing the emerging coding clusters together and in relation to the entire data set. The final stage of data analysis consisted of reporting the results of the previous stages using different categories, as an expression of the manifest content of our UAs. In this article we present the categories of future use, recontact of participants and data sharing in relation to future use because of the way they were linked in terms of implementation and the provision of informed consent. An independent content analysis on the themes of “Genetic Counseling”, “Return of results”, and “IRB-Approved forms for NGS research in mental health” was previously reported [12].

### Identification of targeted subjects and TCPS2 coverage

The research ethics policy applied by the three Canadian research agencies (TCPS2 [10]) was used to frame our content analysis. The subject of future or secondary use of biological material and/or data is discussed extensively throughout the TCPS2 [10], underlining the fact that it is a recurring issue at different stages of the ethical conduct of a research project. It is addressed in the contexts both of research and of the collection of biological materials (including genetic material), as well as for general or particularly vulnerable populations.

## Results

### Characteristics of IC documents

We evaluated if and how secondary use was presented in our UAs. Out of a total of 37 UAs, 33 broached this subject (Table 1a). One in 33 addressed secondary use (Table 1a), but outside the context of genetics. We therefore analyzed 32 secondary-use UAs, classified according to the field of research (Table 1a). The field was derived from the title of the document or the goals of the institution.

### Secondary use

Secondary use (also called “future use” in TCPS2 [10] and in several documents analyzed herein) was mentioned in documents’ main title, main title and subtitles, or in no title at all. In addition it was present in a variety of categories ranging from optional studies to confidentiality and storage of samples, among others (Table 1b).

In our UAs, IRBs suggested different approaches to obtain participant consent for secondary use of their samples or data. Twenty seven UAs (27/32) provided template texts. For 22 of those 27 we also found instructions to researchers on how to approach the issue of secondary use at time of consent. The 5 out of 32 UAs without template texts had instructions to researchers included in IRB documents other than templates.

We found various modalities for secondary use. Participants either had no choice presented, or could choose among a varying number of options on secondary use, for instance that samples will be destroyed, or samples will be banked. Only eight UAs (8/32) provided a clear choice of *no secondary use (future use) at all* (Table 1c).

Four UAs (4/32) foresaw the use of collected samples for *future research in deceased participants*, in accordance with the reference on local regulations (Art 12.1, TCPS2) [10].

The following were present as general information *(not options)* to participants: the possible use of secondary data from linked databases (2/32), while three in 32 explained that because of the fast changes in technology “the potential future use of genetic information is unknown” and so potential future risks derived from such information were also unknown. One UA (1/32) advised researchers that when there were plans to use “genetic testing…to determine eligibility”, that had to be mentioned when discussing secondary use.

According to guidelines set forth in the TCPS2 [10], any plans on secondary use of identifiable data or materials need to be approved by the institutional IRB. We thus checked if our UAs contained any message to be conveyed to participants in this regard, or, alternatively, a reminder to researchers about this requirement. A total of 18 UAs (18/32) presented a text on this subject.

As for the object of secondary use, 19 UAs referred to biological material only (19/32). Secondary use of data only was described in 2 (2/32), while secondary use of both material and data was described in 10 (10/32). One UA (1/32) did not describe the goal of secondary use.

### Sharing data and material related to future use

The TCPS2 [10] requests that researchers provide participants with “a description of the anticipated uses of data” (art 3.2), to help them make an “informed decision” on whether to participate or not in a given research project. In addition, it requests that IRBs receive an explanation of “the full life cycle” of collected information (art 5.3). For “genetic material banks” (art 13.7a), researchers are asked to specify to participants and IRBs the “use of the data and results” as well as the “ethical issues” raised by the future use of data, if the latter is planned (Art 13.7b).

A total of 26 out of 37 UAs discussed the matter of “sharing” (Table 2a). Two of these 26 UAs are included in this group because the subject of sharing was discussed in additional documents, although not in the templates. We analyzed how “sharing” is framed in the UAs to recognize how it might be conveyed to participants. Sharing of data and material was addressed under different headings varying from confidentiality to genetic testing/genetic research, other research or mentioned in the IC template text without any title drawing attention to it.

**Table 2:** **Sharing of material and data**

a: Sharing of material and data			In relation to secondary use				Total
			NA	No	Not explicitly	Yes	
Sharing material and/or data		No	10	1	0	0	11
		Yes	0	5	2	19	26
	Total		10	6	2	19	37
**b: Sharing**			**Material separated from data?**				**Total**
			**Material only**	**NA**	**No**	**Yes**	
		No	0	11	0	0	11
		Yes	7	0	4	15	26
Total			7	11	4	15	37
**c: REB in sharing material/data**			**REB mentioned**			**Total**	
			**NA**	**No**	**Yes**		
Sharing material and/or data		No	11	0	0	11	
		Yes	0	18	8	26	
Total			11	18	8	37	
**d: Particularities on sharing of material and/or data**				**Frequency**	**Percent**	**Valid percent**	**Cumulative percent**
Valid	Data sharing is mentioned on an application/checklist form			1	2.7	2.7	2.7
	Information to researcher			1	2.7	2.7	5.4
	NA			10	27.0	27.0	32.4
	No			22	59.5	59.5	91.9
	Possibilities on sharing samples and or data among private entities and academic institutions			1	2.7	2.7	94.6
	Withdraw of tissue is separated from data			1	2.7	2.7	97.3
	Types of data bases (public/open and close) are defined			1	2.7	2.7	100.0
Total				37	100.0	100.0	

NA: not applicable.

We explored sharing materials and data in relation to secondary (future) use (Table 2a), and if data and materials were considered two separate entities (Table 2b). We found that the matter of “sharing” (material and/or data) was either presented in relation to secondary use (19/26), not definitely related to secondary use (2/26), or, still, not related to secondary use (5/26) (Table 2a). A total of 15 UAs (15/26) discuss the sharing of material independently of sharing data, while the rest mentioned sharing material only (7/26) or sharing material and data as one entity (4/26) (Table 2b). Just eight samples (8/26) discussed IRB involvement in approving the sharing of materials or data (Table 2c). Distinctive comments in our UAs concerning sharing material and/or data are summarized in Table 2d.

### Recontacting participants

Various circumstances can lead investigators to want to recontact participants. The TCPS2 [10] anticipates that if researchers collect identifiable information (Art. 5.6) or samples (Art. 12.4) for which secondary use was approved there is no need to undergo a new consent process. Thus, there would be no need to recontact participants for that purpose. When the purpose of the consent is to bank human biological material, researchers are expected to determine in advance in which circumstances participants would be recontacted, and how (Art. 13.7) [10].

A total of 23 (out of 37) UAs considered the possibility of recontacting participants (Table 3a). Eight UAs (8/37) talked about secondary use but did not mention recontact, whereas only one (1/37) mentioned recontact without discussing future use or any particular reason for doing so.

**Table 3:** **Recontacting participants**

a: Recontacting		In relation of secondary use			Total
		NA	No	Yes	
Recontact	No	1	5	8	14
	Yes	0	1	22	23
Total		1	6	30	37
**b: Reasons for recontacting**		**Frequency**	**Percent**	**Valid percent**	**Cumulative percent**
Valid	Further health data	1	2.7	2.7	2.7
	Future research	4	10.8	10.8	13.5
	Future research related to present project	2	5.4	5.4	18.9
	Future research, additional information	1	2.7	2.7	21.6
	Future research, additional information, family members	1	2.7	2.7	24.3
	Future research, family members	3	8.1	8.1	32.4
	Future research, future use of biological material in genetic research	1	2.7	2.7	35.1
	Future research/future information	1	2.7	2.7	37.8
	Future use of samples	3	8.1	8.1	45.9
	NA	14	37.8	37.8	83.8
	Not specified	1	2.7	2.7	86.5
	Other research projects	1	2.7	2.7	89.2
	Results, recontact	3	8.1	8.1	97.3
	Return results	1	2.7	2.7	100.0
	Total	37	100.0	100.0	
**c: Recontacting_Headings**		**Frequency**	**Percent**	**Valid percent**	**Cumulative percent**
Valid	Do I have any options	1	2.7	2.7	2.7
	Does my child have any options	1	2.7	2.7	5.4
	Open consent	1	2.7	2.7	8.1
	Open consent and document to recontact	1	2.7	2.7	10.8
	What about confidentiality	1	2.7	2.7	13.5
	Who can participate in the study	1	2.7	2.7	16.2
	Confidentiality	1	2.7	2.7	18.9
	Consent	7	18.9	18.9	37.8
	Consent, document to recontact	2	5.4	5.4	43.2
	Future contact/future research/other use	1	2.7	2.7	45.9
	NA	13	35.1	35.1	81.1
	Other research	1	2.7	2.7	83.8
	Possibility of recontacting	1	2.7	2.7	86.5
	Recruitment for future projects	1	2.7	2.7	89.2
	Requirement to researcher	1	2.7	2.7	91.9
	Storage and safekeeping of DNA	1	2.7	2.7	94.6
	Suggested as a box	1	2.7	2.7	97.3
	What about confidentiality	1	2.7	2.7	100.0
	Total	37	100.0	100.0	

NA: not applicable.

We found no consensus on the reasons for recontacting participants in our UAs (Table 3b). Grounds for recontacting were presented in the majority of UAs, from participation in future studies/future research to the return of study results – in only two UAs – and only one referred to recontact in order to obtain additional health information (Table 3b). The other reason for future interaction with participants was contact and recruitment of family members (4/22). Our analysis shows that only one UA specifies that the modality of recontacting needs to be explained by the researcher. The matter of recontacting participants was presented under vary diverse headings (Table 3c). The role of the IRB in approving the approach used for initiating recontact was not covered in any of our UAs.

### Secondary use, sharing material and data and recontacting participants in genetic research studies addressing vulnerable populations

We obtained IRB-approved consent forms for four different kinds of NGS studies - on mental health and brain disorders, in adults as well as minors. The majority of them also provided for the recruitment of non-related healthy volunteers. Only one of the consent forms did not discuss secondary use or recontacting participants, and samples and data were to be used exclusively for the issue that was prompting the study. Sharing of both material and data was clearly explained for the purpose of that particular research project.

The other three studies conveyed the matter of secondary use under different headings, similarly to what we present in the general analysis. One document referred to secondary use for identical and related conditions, while the rest provided participants with an array of choices. The role of IRBs in overseeing future use was discussed in only one document, in which participants were also offered additional choices on secondary use. Sharing of material and data were clearly explained as separate entities and options for participants in this matter were present in some cases. Use of various databases was explained. Recontacting participants was anticipated in all three projects, so as to obtain more samples, obtain more information, or for secondary use. Interestingly, one anticipates secondary use in the same research area but using a different methodology.

Request for permission to extend participation to family members was present in all mental health and brain disorders research projects. As for the descriptions of genetic methodology used in these studies, one explains briefly how a genetic marker could be detected while another one states that the researchers will be using more advanced genotyping technologies. However, none provided further details on the technical aspects of the sequencing procedures. We did find a more complete explanation on sharing material and data and the use of databases.

## Discussion

### Analysis of findings

There is a high percentage of UAs that mentioned the secondary use of biological material and/or data. The subject of secondary uses was not broached in a systematic way from one UA to another. We understand that these different approaches to this subject could probably reflect individual decisions by IRBs as to vocabulary and preferred ways of communicating with participants. Different IRBs conceive the ethical issues underlying secondary use differently (e.g., confidentiality, access to samples). However, we understand that the use of explicit terms could certainly contribute to identify the subject matter and, thus, help participants clearly distinguish explanations about the secondary (future) use of their genetic material and data. Modalities on secondary uses of collected material and information also diverged. Some UAs classified their own options as closed, open or tiered. The majority of the UAs discussed the secondary use of material more than that of data. The number of UAs was insufficient for comparative purposes between objects of research and modalities of secondary use.

Sharing what was collected (material, data, or both) was also framed separately, like its secondary use. We found that sharing was related most often to secondary use, and most of the UAs referred to sharing both material and data. Again, as to secondary use, we noted that information on the sharing of genetic sequencing data in a manner completely independent of the material from which it originated was absent.

In over half of our UAs the reason to recontact a participant was to obtain consent for secondary use. In a minority of cases it was to return results or to update health information. As NGS technologies have a high likelihood of unveiling causative and predictive information about clinical conditions related or unrelated to the primary goal of a study, return of results in this context is a dominant subject that could be perceived both as carrying a risk and presenting a benefit to participants. We noted that not much consideration was given to the need of recontacting participants to update health information. Such updates would contribute markedly to understanding new findings in genetic research. As genetic sequencing becomes more common in research, recruitment of participants based on earlier genetic research results becomes of greater importance. Interestingly, there wasn’t any reference to such a possibility, although one UA reminded researchers to tell participants if recruitment was based on genetic testing.

The role of IRBs in overseeing secondary use was clear in the majority of the documents. However, their role was not explicitly stated in regard to sharing samples and/or data or on recontacting participants. We consider that the role of the IRB should deserve equal consideration in these three undertakings: secondary use, sharing of material and data and recontacting participants.

The emergence of NGS technologies creates the need for a more complex and thorough explanation of new(er) concepts to study participants, such as sharing information between researchers to recruiting participants based on earlier genetic results with an undefined clinical significance. Clarification of these issues aims to uphold the principles of autonomy and beneficence: autonomy of research participants in deciding how they want their DNA samples (and the information derived from them) to be handled, and beneficence in avoiding any harm due to possible misuse of samples and data, all the while maximizing possible benefits to them, and to society as a whole. Our content analysis of a Canadian sample of template texts and accompanying IRB instructions addressing the consent process for genetic studies showed compliance with the requirements set forth by the TCPS2. We found that, in Canada, there were not many gaps to bridge between current regulations and the evolving needs regarding informed consent due to the increasingly widespread use of NGS in relation to the three subjects discussed in this article. There is an amount of specificity that should be expected for any given genetic project and that is the result of the interaction between the investigators and any IRB, and that was missing in a majority of the documents used in our analysis. Yet, although based on a small set, when comparing the IRB-approved consent forms for brain disorders and the templates used to prepare these documents, there were relatively few differences.

Lastly, current literature discusses the importance of stronger safeguards for vulnerable populations, in particular those affected by mental health or brain disorders [8, 9]. Cognitive limitations in individuals affected with mental health disorders, and the likelihood of therapeutic misconceptions in individuals with mental health and brain disorders such as autism spectrum disorders and their immediate family have been reported and discussed [8, 9, 11]. However, we didn’t find any particular language addressing these participants specifically in the subset of IRB-approved forms, neither in the subjects discussed in this work nor elsewhere on the consent forms. Mental health and brain disorders carry additional risks of disclosure and stigma for probands and their immediate family. It is also true that anyone from the general population currently participating in any type of genetic research could find out in the future that they belong to a particularly vulnerable population. In this respect, such individuals should be entitled to the same protection as those entering a study with “full” knowledge of their condition. The difference, then, could reside in the consent process itself, which could include more complete explanations and the recourse to additional decision-making tools during the consent process [16]. In any case, the correct process for obtaining consent from any population should include close monitoring of the informed consent process, which means more than what the TCPS2 and present IRB forms currently provide.

### Bridging the gaps with current needs

Based on the results of the present study and on the reported impact of NGS in genetic research related to secondary use, sharing of data and material and recontacting participants, [17–20] we deem important that the following concepts be taken into consideration by ethics committees and researchers so as to provide a fully informed consent to research participants:

- In genetic studies, there must be a clear distinction between the secondary use of biological material and data: while DNA samples may well not be shared, sharing of the generated genetic data can become unavoidable [20].
- We consider that changes in technology that have the potential to impact participants’ preferences should be brought to their attention. Similarly to the context of clinical trials addressed in the TCPS2 [10], when there is new information on the risk/benefit ratio for research participants that might modify their decision to stay in the study, this new knowledge must be disclosed. The means of doing so (i.e., general notifications, consent form addendums) would greatly depend on the feasibility of any follow-through on the part of IRBs and researchers.
- De-identified DNA is becoming more easily linked to individuals or groups of individuals even when using exclusively public databases [21], and DNA could be considered a personal identifier in and of itself [22]. Simply informing research participants about the possibility that their genetic data might be added to public databases, as per the TCPS2 [10], is then no longer sufficient. When data linkage of two or more anonymous sets of information or human biological materials is planned, an IRB review should be required for any population addressed in the study, and not only for the more vulnerable (i.e., aboriginal communities [10]). In addition to current literature [23], we propose that a layered consent process for genetic data sharing would be appropriate.
- Current technological approaches to genetic studies impact the communication between researchers and research participants, generating further reasons to remain in contact. To define the grounds that promote maintaining contact with participants during the earlier stages of a research project, we share the position adopted by Beskow et al. on advising participants that “recontacting will occur”, reminding participants that the option to refuse to be informed of the reason that prompts any recontact should always be open [24]. All communication, as recommended in the TCPS2 [10], should be dealt with by individuals or institutions known to participants [24].

## Conclusions

Our analysis of Canadian consent template texts on secondary use, sharing of data and material, and recontacting participants, in the context of genetic research, shows that only minor, targeted modifications would be required to increase the protection of participants in NGS research.

The ethical oversight of research projects evolves to keep pace with ethical challenges in the face of unpredictable technological changes, ensuring concurrent protection of research participants. Examples of changes in ethical oversight of research include multicenter reviews [24], as well as current proposals to reform the ethical management framework for low-risk, randomized research projects [2]. A comparison could be drawn between these examples and the changes brought about in the ethical management of research projects using NGS technologies. The issues of secondary use of genetic material or data, how the latter are shared, the need for ongoing communication between researchers and participants, in addition to managing, for instance, the return of genetic results, are all common challenges to the genetic research community at large. We thus believe that the results of our analysis could be valuable to improve the quality of research participants’ information and consent documentation, be it for current or upcoming genetic studies, in Canada and elsewhere.

## References

[1] Hayden EC (2012). Informed consent: a broken contract. Nature.

[2] Faden RR, Beauchamp TL, Kass NE (2014). Informed consent, comparative effectiveness, and learning health care. N Engl J Med.

[3] Caulfield T, McGuire AL, Cho M, Buchanan JA, Burgess MM, Danilczyk U, Diaz CM, Fryer-Edwards K, Green SK, Hodosh MA, Juengst E, Kaye J, Kedes L, Knoppers BM, Lemmens T, Meslin EM, Murphy J, Nussbaum RL, Otlowski M, Pullman D, Ray PN, Sugarman J, Timmons M (2008). Research ethics recommendations for whole-genome research: consensus statement. PLoS Biol.

[4] Henderson GE (2011). Is informed consent broken?. Am J Med Sci.

[5] Rotimi CN, Marshall PA (2010). Tailoring the process of informed consent in genetic and genomic research. Genome Med.

[6] Bredenoord AL, Onland-Moret NC, Van Delden JJ (2011). Feedback of individual genetic results to research participants: in favor of a qualified disclosure policy. Hum Mutat.

[7] Kronenthal C, Delaney SK, Christman MF (2012). Broadening research consent in the era of genome-informed medicine. Genet Med.

[8] Groisman IJ, Mathieu G, Godard B (2012). Use of next generation sequencing technologies in research and beyond: are participants with mental health disorders fully protected?. BMC Med Ethics.

[9] Mathieu G, Groisman IJ, Godard B (2013). Next generation sequencing in psychiatric research: what study participants need to know about research findings. Int J Neuropsychopharmacol.

[10] Canadian Institutes of Health Research Natural Sciences and Engineering Research Council of Canada, & Social Sciences and Humanities Research Council of Canada (2010). Tri-Council Policy Statement: Ethical Conduct for Research Involving Humans (TCPS 2).

[11] Baret L, Godard B (2011). Opinions and intentions of parents of an autistic child toward genetic research results: two typical profiles. Eur J Hum Genet.

[12] Egalite N, Groisman IJ, Godard B (2014). Genetic counseling practice in next generation sequencing research: implications for the ethical oversight of the informed consent process. J Genet Couns.

[13] Vaismoradi M, Turunen H, Bondas T (2013). Content analysis and thematic analysis: implications for conducting a qualitative descriptive study. Nurs Health Sci.

[14] Sparkes AC, Holloway I (2005). Narrative Analysis: Exploring the Whats and Hows of Personal Stories. Qualitative Research in Health Care.

[15] Grbich C (2007). Qualitative Data Analysis: An Introduction.

[16] Brehaut JC, Saginur R, Elwyn G (2009). Informed consent documentation necessary but not sufficient. Contemp Clin Trials.

[17] Kaye J, Boddington P, de Vries J, Hawkins N, Melham K (2010). Ethical implications of the use of whole genome methods in medical research. Eur J Hum Genet.

[18] McGuire AL, Caulfield T, Cho MK (2008). Research ethics and the challenge of whole-genome sequencing. Nat Rev Genet.

[19] Ries NM, LeGrandeur J, Caulfield T (2010). Handling ethical, legal and social issues in birth cohort studies involving genetic research: responses from studies in six countries. BMC Med Ethics.

[20] Tabor HK, Stock J, Brazg T, McMillin MJ, Dent KM, Yu JH, Shendure J, Bamshad MJ (2012). Informed consent for whole genome sequencing: a qualitative analysis of participant expectations and perceptions of risks, benefits, and harms. Am J Med Genet A.

[21] Gymrek M, McGuire AL, Golan D, Halperin E, Erlich Y (2013). Identifying personal genomes by surname inference. Science.

[22] Rodriguez LL, Brooks LD, Greenberg JH, Green ED (2013). Research ethics. The complexities of genomic identifiability. Science.

[23] McGuire AL, Oliver JM, Slashinski MJ, Graves JL, Wang T, Kelly PA, Fisher W, Lau CC, Goss J, Okcu M, Treadwell-Deering D, Goldman AM, Noebels JL, Hilsenbeck SG (2011). To share or not to share: a randomized trial of consent for data sharing in genome research. Genet Med.

[24] Greene SM, Geiger AM (2006). A review finds that multicenter studies face substantial challenges but strategies exist to achieve Institutional Review Board approval. J Clin Epidemiol.

[CR25] The pre-publication history for this paper can be accessed here:http://www.biomedcentral.com/1472-6939/15/80/prepub

